# Plant diversity influenced gross nitrogen mineralization, microbial ammonium consumption and gross inorganic N immobilization in a grassland experiment

**DOI:** 10.1007/s00442-020-04717-6

**Published:** 2020-07-31

**Authors:** Soni Lama, Andre Velescu, Sophia Leimer, Alexandra Weigelt, Hongmei Chen, Nico Eisenhauer, Stefan Scheu, Yvonne Oelmann, Wolfgang Wilcke

**Affiliations:** 1 Institute of Geography and Geoecology, Karlsruhe Institute of Technology (KIT), Reinhard-Baumeister-Platz 1, 76131 Karlsruhe, Germany; 2 Institute of Biology, Leipzig University, Johannisallee 21, 04103 Leipzig, Germany; 3 German Center for Integrative Biodiversity Research (iDiv) Halle-Jena-Leipzig, Deutscher Platz 5e, 04103 Leipzig, Germany; 4 JF Blumenbach Institute of Zoology and Anthropology, University of Göttingen, Berliner Strasse 28, 37073 Göttingen, Germany; 5 Geoecology, University of Tübingen, Rümelinstrasse 19-23, 72070 Tübingen, Germany

**Keywords:** N cycling, Biodiversity, C:N ratio, ^15^N isotopic pool dilution, The Jena Experiment

## Abstract

Gross rates of nitrogen (N) turnover inform about the total N release and consumption. We investigated how plant diversity affects gross N mineralization, microbial ammonium (NH_4_^+^) consumption and gross inorganic N immobilization in grasslands via isotopic pool dilution. The field experiment included 74 plots with 1–16 plant species and 1–4 plant functional groups (legumes, grasses, tall herbs, small herbs). We determined soil pH, shoot height, root, shoot and microbial biomass, and C and N concentrations in soil, microbial biomass, roots and shoots. Structural equation modeling (SEM) showed that increasing plant species richness significantly decreased gross N mineralization and microbial NH_4_^+^ consumption rates via increased root C:N ratios. Root C:N ratios increased because of the replacement of legumes (low C:N ratios) by small herbs (high C:N ratios) and an increasing shoot height, which was positively related with root C:N ratios, with increasing species richness. However, in our SEM remained an unexplained direct negative path from species richness to both N turnover rates. The presence of legumes increased gross N mineralization, microbial NH_4_^+^ consumption and gross inorganic N immobilization rates likely because of improved N supply by N_2_ fixation. The positive effect of small herbs on microbial NH_4_^+^ consumption and gross inorganic N immobilization could be attributed to their increased rhizodeposition, stimulating microbial growth. Our results demonstrate that increasing root C:N ratios with increasing species richness slow down the N cycle but also that there must be additional, still unidentified processes behind the species richness effect potentially including changed microbial community composition.

**Electronic supplementary material:**

The online version of this article (10.1007/s00442-020-04717-6) contains supplementary material, which is available to authorized users.

## Introduction

Biodiversity loss has raised concern over the consequences for ecosystem functioning (Isbell et al. 2011; Cardinale et al. 2012; Meyer et al. 2016; Weisser et al. 2017). Plant diversity is essential for maintaining a variety of ecosystem functions (Hector et al. 1999; Loreau et al. 2001; Tilman et al. 2001; Roscher et al. 2005; Cardinale et al. 2012), including nitrogen (N) cycling (Spehn et al. 2005; Fornara and Tilman 2009; Oelmann et al. 2011; Reich et al. 2012; Rosenkranz et al. 2012). Biodiversity experiments have mainly reported increased community productivity with increasing plant diversity (Tilman et al. 2001; Spehn et al. 2005; Marquard et al. 2009). A potential reason for the positive species richness–biomass production relationship might be complementarity effects in species-rich mixtures (Hooper and Vitousek 1998; Fargione et al. 2007; Reich et al. 2012). Complementarity effects occur when more-diverse communities increase their performance above the expected performance of monocultures through acquiring more nutrients and using available light and space more exhaustively (Hooper and Vitousek 1997; Naeem 2002). Complementarity also includes the process of facilitation, for example by legumes, which increase the nutrient availability for neighboring plants via N_2_ fixation (Fargione et al. 2007). However, in about 1/3 of the reported experiments, complementarity effects did not increase productivity likely because of the selection for more competitive but less productive species at higher diversity (Cardinale et al. 2011). Furthermore, sampling effects can arise in biodiversity experiments, if the probability of sampling dominant species increases in high diversity levels (Huston 1997; Loreau et al. 2001). To prevent such sampling effects and to be able to detect other mechanisms behind biodiversity–ecosystem functioning relationships, biodiversity experiments need to be carefully designed (Loreau et al. 2001; Roscher et al. 2004). Increasing plant diversity modifies resource availability for soil microbial communities (Zak et al. 2003), which mineralize organic matter and enhance nutrient release by litter decomposition. Plant species differ in their biochemical composition providing an incentive for microbes to derive different resources from different litter types (Gartner and Cardon 2004). This might result in altering overall decomposition rates of mixtures relative to the cumulative composition of individual litter species (Gessner et al. 2010). Jewell et al. (2015) reported a faster decomposition rate of monospecific litter in its environment of origin but not of mixed litter. Although it has been reported that complementarity can result in high plant productivity and N uptake, it is uncertain if the changes in plant diversity affect microbial N dynamics.

Plants play a vital role in ecosystem N cycling, because plants assimilate this essential nutrient to produce biomass, which is returned as aboveground and belowground litter to soil, where it is decomposed, thereby releasing the N back into the soil solution (Knops et al. 2002; Vitousek et al. 2002). Individual plant species can positively affect the N cycle in soil by the activity of plant roots (e.g., fine root turnover, root exudation; Clarholm 1985; Cadisch and Giller 1997) and by regulating the quality of plant litter (measured as C:N ratios, Aerts et al. 1992; Van Vuuren et al. 1993; Abbas et al. 2013; Guiz et al. 2015). Plant species that host N_2_-fixing bacteria can change N cycling by improving the N availability to other co-occurring species (Mulder et al. 2002; Spehn et al. 2005). Another way in which plant species may affect rates of N cycling is through their association with mycorrhizal fungi, which enhance the ability of plants to acquire nutrients (Hobbie 1992).

Because of the importance of N in all ecosystems and the marked impact of human activities on the N cycle, N and its transformations have received a great deal of attention. The supply rate of N to the plant and microbe community depends largely on gross N mineralization, which is described as the total N transformed from organic N to mineral N forms (NH_4_^+^, NO_3_^−^) by microorganisms in soil over a period of time that can be readily taken up by plants and microbes. Microbial ammonium consumption refers to the microbial assimilation of NH_4_^+^ plus the gross nitrification. Gross inorganic N immobilization is the process of converting inorganic forms of N by microbes and other soil heterotrophs to organic N forms. Net N mineralization refers to the gross mineralized N minus the quickly microbially consumed N. Net ammonification is the difference between gross N mineralization and microbial NH_4_^+^ consumption, and net nitrification is that between gross nitrification and NO_3_^−^ immobilization.

Hobbie (1992) reported that the strong relationship between litter quality and gross N mineralization rates might indicate that gross N mineralization rates are determined by the quality of litter input. This was corroborated by the results of Van der Krift et al. (2001) who reported that the quantity and quality of plant litter determine N release in soil. Because the quantity and quality of soil organic matter results from decomposition of aboveground and belowground biomass and rhizodeposition, there is also a link between soil organic matter quantity and quality and N supply via net N mineralization (Benbi and Richter 2002; Hobbie 2015). Soil microbes release nutrients by mineralization of soil organic matter and decomposition of fresh litter. Resource availability for soil microorganisms or microbial uptake is also regulated by litter decomposition (Smith and Paul 1990). Plant litter varies in chemical composition; therefore, changes in plant communities could alter the production and types of organic compounds in soil, thereby controlling the composition and function of microbial communities (Zak et al. 2003). Moreover, environmental conditions, such as soil pH, soil moisture, soil temperature, and soil texture influence gross N mineralization by changing microbial biomass or activity associated with substrate availability (Booth et al. 2005; Wang et al. 2016; Zhang et al. 2016).

In particular, root C:N ratios explained high amounts of variance in gross N mineralization rates in soil (Fornara et al. 2011). Litter with high C:N ratios is considered as low quality, whereas litter with low C:N ratios is considered as high quality. Previous studies showed that high root C:N ratios have a strong negative effect on gross N mineralization (Silver and Miya 2001; Fornara et al. 2011). There is increasing evidence that root decomposition may be more important than aboveground plant biomass decomposition for organic matter formation and the associated N stocks in soil (Rasse et al. 2005; Kramer et al. 2010). The work of Ruppenthal et al. (2015) has even suggested that root litter is the dominant source of soil organic matter. Fornara et al. (2011) reported that gross N mineralization rates are mainly driven by changes in C and N concentrations of soil organic matter. Consequently, root decomposition could be the major source of N released by mineralization in soil. This is further supported by Abbadie et al. (1992), who found indirect evidence that the most assimilated N originated from root decay in African grasslands.

Plant diversity influences several N-transformation processes in soil via plant uptake of N and modifications of ecosystem properties like microbial community or biomass production (Hooper and Vitousek 1998; Spehn et al. 2005; Weisser et al. 2017). Previous biodiversity studies in grasslands have mainly reported positive relationships between plant species richness and both gross and net N mineralization rates (e.g., West et al. 2006; Rosenkranz et al. 2012; Mueller et al. 2013) and net nitrification rates in the presence of legumes (Scherer-Lorenzen et al. 2003). Rosenkranz et al. (2012) found that the increasing topsoil water content with increasing plant species richness was the main factor underlying positive effects of plant species richness on net N mineralization rates in the Jena Experiment, the same experimental site as in this study. Another plant diversity experiment showed that positive effects of plant diversity on net N mineralization rates were driven by increased N concentrations in roots (Mueller et al. 2013). In an isotope dilution experiment in the laboratory using soil samples from the BioCON experiment in the North American prairie, gross N mineralization rates increased with increasing plant species richness because of greater microbial activity (West et al. 2006). In addition, net N mineralization rates decreased and N immobilization rates increased at higher species diversity (West et al. 2006). However, the incubation experiment was conducted inside a laboratory, which could not necessarily be directly comparable to field conditions (e.g., because of cold storage of the samples before lab incubation, controlled incubation temperature, and optimum nutrient supply; Arnold et al. 2008). To our knowledge, no study has been reported that investigated plant diversity effects on microbial NH_4_^+^ consumption and on gross inorganic N immobilization rates in situ.

Besides plant species richness, the presence or absence of specific plant functional groups can affect N cycling in grassland ecosystems (Scherer-Lorenzen et al. 2003; Oelmann et al. 2007; Dybzinski et al. 2008; Fornara and Tilman 2009; Fornara et al. 2011; Leimer et al. 2015). Legumes constitute a distinct functional group in grasslands because of their ability to fix atmospheric N via symbiotic root microorganisms (Spehn et al. 2002; Marquard et al. 2009). Mulder et al. (2002) reported that non-leguminous plants depend on N_2_ fixed by legumes to counter-balance the declining soil N availability in unfertilized (near-) natural ecosystems. Therefore, many studies concluded that with an increased legume biomass, there is a larger plant-available N pool in the soil (Spehn et al. 2002; Booth et al. 2005; Scherer-Lorenzen 2008). This larger plant-available N pool can originate from increased gross N mineralization of N-rich legume litter. Besides legumes, grasses were also found to influence gross N mineralization. Oelmann et al. (2007) reported that the presence of grasses decreased mineral N pools in soil compared to plant communities without grass species because of their dense and extensive rooting system. This extensive rooting system is efficient in taking up soil N and thus can reduce mineral N pools in soil (Oelmann et al. 2007).

The objectives of our study were (i) to investigate if plant species richness, functional group richness or the presence/absence of individual functional groups (together termed plant diversity) affect gross N mineralization, microbial NH_4_^+^ consumption and gross inorganic N immobilization rates and (ii) to determine the underlying controls responsible for the potential relationships. We hypothesized that there was a positive effect of plant species richness on gross N mineralization rates because of the known positive relationship between plant species richness and microbial activity in the Jena Experiment (Strecker et al. 2016). Second, we expected an increasing microbial NH_4_^+^ consumption and gross inorganic N immobilization with increasing plant species richness because of the higher N demand and the tighter N cycling in species-rich than in species–poor plant mixtures. Thirdly, we hypothesized that the presence of legumes increased gross N mineralization, microbial NH_4_^+^ consumption and gross inorganic N immobilization because of the smaller C:N ratio of litter in plant mixtures containing legumes compared to plant mixtures without legumes (Chen et al. 2017). Although our focus was on gross N turnover rates, we additionally calculated the rates of net mineralization and its components net ammonification and net nitrification and analyzed their relationship with plant diversity.

## Materials and methods

### Study site

Our study was part of the Jena Experiment (www.the-jena-experiment.de), a long-term grassland diversity experiment established in 2002 (Roscher et al. 2004; Weisser et al. 2017). The site had been used as arable land for at least 40 years before the establishment of the Jena Experiment. The experimental site is located on the floodplain of the river Saale in Jena, Germany (50°55′ N, 11°35′ E; 130 m above sea level). Mean annual air temperature is 9.9 °C, and mean annual precipitation amounts to 610 mm (1980–2010, Hoffmann et al. 2014). The soil at the site is classified as Eutric Fluvisol developed from 2-m thick loamy fluvial sediments (IUSS Working Group WRB 2014). The soil texture ranges from sandy loam close to the river to silty loam with increasing distance from the river. The mean bulk density of the topsoil (0–5 cm) of the experimental plots is 1.18 ± 0.1 g cm^−3^; varying little from 1.21 ± 0.1 g cm^−3^ in Block I with the lowest clay content to 1.17 ± 0.1 g cm^−3^ in Block IV with the highest clay content. The experimental site is mown twice and weeded three times a year to maintain the designed diversity levels. The biomass was removed after mowing/weeding. This management mimics a typical use of semi-natural species-rich mesophilic grassland as hay meadow (Roscher et al. 2004). A major aim of the Jena Experiment is to explore the effect of biodiversity on nutrient cycling and trophic interactions.

A detailed description of the experimental design is provided in Roscher et al. (2004). The main experiment consists of 82 plots (20 m × 20 m) in four blocks to account for the systematic change in soil texture perpendicular to the river with a factorial design (as far as possible) of different levels of plant species richness (1, 2, 4, 8, 16, and 60) and 1–4 functional groups (grasses, legumes, small herbs, and tall herbs). The mixtures were randomly drawn from a pool of 60 species representing typical Central European mesophilic grasslands. All the 16 species of grasses are perennial except *Bromus hordeaceus* L. Each level of species richness was replicated on 16 plots except for the 16 and 60 species richness levels, which were only replicated on 14 and 4 plots, respectively. Since there were only four replicates of the 60-plant species mixture, we excluded them from our data analyses (which reduced the number of considered plots to 78). Of these 78 plots, we lost two because of errors during the laboratory analyses. Those two plots (B2A08 and B4A02) were sown with a species richness level of 2 and 16 and functional group richness of 2 and 3, respectively. Another two plots (B1A09 and B4A03), both monocultures, were abandoned due to their poor performance (i.e., extremely low target species cover). Therefore, our final analyses were based on 74 plots.

### Isotope pool-dilution experiment

We used the isotope pool-dilution method in a field incubation experiment to determine the rates of gross N mineralization in soil (Davidson et al. 1991). We labeled the soil NH_4_^+^ pool with 98 at% ^15^N as NH_4_Cl. While unlabeled N from the organic pool is mineralized to NH_4_^+^ by microorganisms, the ^15^N enrichment of the NH_4_^+^ pool is diluted. The method of Davidson et al. (1991) is based on several assumptions which are valid for short incubation periods of up to 24 h. According to these assumptions, (1) there is no or only negligible isotope discrimination by microorganisms during the incubation period, so that the consumption of NH_4_^+^ alters the pool size, but not the isotope ratio of the pool, (2) the turnover rates are constant, and (3) no N re-mineralization occurs, so that the assimilated ^15^N is not returned to the labeled pool.

A disturbed soil sample was taken to determine the natural ^15^N abundance and 1 M KCl-extractable mineral N (NH_4_^+^-N and NO_3_^−^ -N) concentrations on each plot before starting the experiment. We performed the field experiment and collected soil samples in April 2011. Two pairs of stainless steel cores (*Ø* = 56 mm, *h* = 41 mm, *V* = 100 cm^3^) were taken from within the 0–5 cm layer of the soil of each plot (one pair for each time step, t1 and t2), closed at the bottom side with a polyethylene lid to prevent leaching losses and immediately reinserted. We averaged the two cores for each time step for ^15^N isotopic analysis to improve plot representativity. The soil samples in the cores were labeled with a NH_4_Cl solution (5 mg L^−1^ N, 98 at% ^15^N) using a high-precision, digital dispenser (Brand, Wertheim, Germany) coupled to a side-port needle, which injected the solution horizontally to ensure a homogeneous distribution of the 5-mL label within the cores. For every core, the injections were uniformly distributed at five points, each point receiving 1 mL of the tracer solution. In total, 25 µg N (98 at% ^15^N) were added as label to each core, which corresponds to less than 2 percent of the NH_4_-N concentration in the soil at the time of the experiment.

To account for abiotic NH_4_^+^ fixation, ensure the ^15^N enrichment and calculate tracer recoveries, one pair of the soil cores was removed from the soil after 15 min (t1) and the remaining soil cores after 24 h (t2) to calculate the ^15^N pool dilution after the field incubation. Soil samples from shortly before the pool dilution experiment and from t1 and t2 of the experiment were shaken with 1 M KCl solution for one hour shortly (< 2 h) after sampling next to the field site to extract NH_4_^+^ and NO_3_^−^ and then filtered through ash-free paper filters (no. 595, Schleicher & Schuell, Dassel, Germany, pore size 4–7 μm). The extracts were immediately frozen at – 20 °C and transported in frozen state to the laboratory for further chemical analyses.

The concentrations of NH_4_-N and NO_3_-N in the 1 M KCl extracts were measured by high-resolution colorimetric detection using a continuous flow analyzer (CFA Autoanalyzer 3 h, Seal Analytical GmbH, Norderstedt, Germany). We used the micro-diffusion method (Stark and Hart 1996) to determine the ^15^N/^14^N isotope ratios of NH_4_^+^ in the soil extracts. In the micro-diffusion method, NH_4_^+^ is volatilized as NH_3_ by increasing the pH to > 9.5 with MgO. The released NH_3_ was then collected on an acidified (2.5 M NaHSO_4_) filter disk enclosed in a polytetrafluoroethylene (PTFE) envelope, where it reacted back to NH_4_^+^. The N isotope ratios were determined with an Elemental Analyzer (EA 1110, Carlo Erba Instruments, Milan, Italy) coupled to an isotope-ratio mass spectrometer (MAT Delta Plus, Thermo Finnigan, Bremen, Germany) at the Stable Isotope Center, University of Göttingen. Ten replicate measurements of in-house standard reference material [^15^N-(NH_4_)_2_SO_4_] resulted, on average, in 98.4 ± 1.6% of the true value, indicating a high accuracy of our measurements. The error of ± 1.6% is the average deviation from the true value. Precision of the ^15^N measurements was ± 0.002 at% (*n* = 10).

### Plant community and soil properties

Aboveground (shoot) biomass was harvested in May 2011 prior to mowing. Plants were clipped at 3 cm above ground level within the harvesting area of two replicate 20 cm × 50 cm subplots per plot. Plant material was sorted into sown species, weeds, and dead aboveground biomass. Biomass of each sown species was determined after drying at 70 °C for at least 48 h (Weigelt et al. 2010). For shoot C:N ratio analysis, all the plant material from one plot was pooled together to obtain a representative value for the plant community of the respective plot. A small subsample of this material was milled to fine powder using a ball mill (MM 400, Retsch GmbH, Haan, Germany) and up to 5 mg from each plot was used for C and N analysis (Flash EA 112, Thermo Fisher, Milan, Italy). Shoot height (regenerative shoot height, i.e., soil surface to highest flower) was measured on five individual plants (without stretching the plants) every meter along a 5-m transect in the central area of the plots (61 m^2^) using a ruler.

For the analysis of the root C:N ratio, community roots were collected in September 2013 per plot. The root C:N data were not available for 2011, so we used the data of the nearest possible date. Root biomass was sampled originally for a root decomposition experiment, where the C:N ratio was used as explanatory variable for litter quality (Chen et al. 2017). To minimize disturbance of the experimental plots, we limited larger soil cores (40 × 15 × 20 cm) to plots with low standing root biomass and took smaller soil cores (20 × 10 × 20 cm), where standing root biomass was sufficiently high to provide enough fine root material. Sampling depth was always 20 cm covering the main rooting horizon, where on average 90% of community standing root biomass in the Jena Experiment plots can be found (Chen et al. 2017). Roots were collected, cleaned and sorted to fine (< 2 mm) and coarse roots after washing. Fine roots were oven-dried at 65 °C and ground with a ball mill (MM 400, Retsch GmbH, Germany) and analyzed for total C and N concentrations using an elemental analyzer (Flash 2000, ThermoFisher Scientific Inc, Waltham, MA, USA). Studies have found that fine roots are more active and decompose faster than coarse roots in forest ecosystems (Brunner and Godbold 2007; Lukac 2012; Zhang and Wang 2015). Therefore, we expected similar differences between fine and coarse roots in grasslands. Additionally, although variable among communities, root biomass data at the Jena Experiment showed that fine roots made up on average 84% of the total standing root biomass (0–30 cm).

To determine the concentrations of organic C and total N in soil, five soil samples per plot (0–5 cm) were taken in 2011. All replicates were combined and homogenized. Soil samples were dried at 40 °C and sieved (< 2 mm). The dried samples were ground using a ball mill. An aliquot of these samples was analyzed for total C and N concentrations by an elemental analyzer (vario Max CN, Elementar Analysensysteme GmbH, Langenselbold, Germany). Inorganic C concentrations were determined by elemental analysis after burning the organic carbon at 450 °C in a muffle furnace. Organic C concentrations were calculated by subtracting inorganic C concentrations from total C concentrations.

We used mean microbial biomass C data from the 4 years prior to our experiment (2007–2010, i.e., Phase 2 in Strecker et al. 2016). Microbial biomass C showed a strong temporal variation in the Jena Experiment depending on the microclimatic conditions, which resulted from weather conditions and related plant growth and thus was aggregated to different phases by Strecker et al. (2016). We used Phase 2 data, because we expected it to best represent the microbial biomass conditions that prevailed during our in-situ experiment. For the measurement of soil microbial biomass, soil samples were taken with a steel corer (5 cores per plot, depth 5 cm, diameter 5 cm) and sieved. Microbial biomass C of approximately 5 g soil (fresh weight) was measured using an O_2_-microcompensation apparatus (Scheu 1992). Substrate-induced respiration was calculated from the respiratory response to D-glucose for 10 h at 22 °C (Anderson and Domsch 1978). Glucose was added according to preliminary studies to saturate the catabolic enzymes of microorganisms (4 mg g^−1^ dry weight solved in 400 µL deionized water). The mean of the lowest three readings of O_2_-consumption values within the first 10 h was taken as maximum initial respiratory response (MIRR; [µL O_2_ g^−1^ dry soil h^−1^]) and microbial biomass (µg C g^−1^ dry soil) was calculated as 38 × MIRR (maximum initial respiratory response Eisenhauer et al. 2010).

The microbial C:N ratio of 38 plots (Blocks 1 and 2 only) was determined from the data of microbial biomass C and N, which was measured using chloroform fumigation extraction. Two samples of 7 g soil were taken from each plot, one was fumigated with chloroform vapor for 24 h and the other was not fumigated. Both, the fumigated and non-fumigated samples were extracted with 40 mL 0.5 M K_2_SO_4_ by shaking for 30 min. Total C and N concentrations in the extracts were analyzed by dry combustion in a DIMA-TOC 100 Analyzer (Dimatec, Essen, Germany). Microbial biomass C was calculated as (total C in fumigated soil – total C in non-fumigated soil)/0.45 (Wu et al. 1990). Likewise, microbial biomass N was calculated as (total N in fumigated soil – total N in non-fumigated soil)/0.54 (Brookes and Landman 1985).

### Calculations and statistical analyses

Rates of gross N mineralization, microbial NH_4_^+^ consumption, gross inorganic N immobilization, net N mineralization and its components net ammonification and net nitrification were calculated using Eqs. 1–6, respectively. Equations 1–4 and 6 are from Hart et al. (1994) and Eq. 5 is from Rosenkranz et al. 2012:

1 $$m = \frac{{\left[ {{\text{NH}}_{4}^{ + } } \right]_{t1} - \left[ {{\text{NH}}_{4}^{ + } } \right]_{t2} }}{t} \times \frac{{\log \left( {\frac{{{\text{APE}}_{t1} }}{{{\text{APE}}_{t2} }}} \right)}}{{{\log}\left( {\frac{{[{\text{NH}}_{4}^{ + } ]_{t1} }}{{[{\text{NH}}_{4}^{ + } ]_{t2} }}} \right)}}$$

2 $$c = m - \frac{{\left[ {{\text{NH}}_{4}^{ + } } \right]_{t1} - \left[ {{\text{NH}}_{4}^{ + } } \right]_{t2} }}{t}$$

3 $$i = m - n$$

4 $${\text{nm}} = \frac{{\left[ {{\text{NH}}_{4}^{ + } + {\text{NO}}_{3}^{ - } } \right]_{t2} - \left[ {{\text{NH}}_{4}^{ + } + {\text{NO}}_{3}^{ - } } \right]_{t1} }}{t}$$

5 $${\text{na}} = \frac{{\left[ {{\text{NH}}_{4}^{ + } } \right]_{t2} - \left[ {{\text{NH}}_{4}^{ + } } \right]_{t1} }}{t}$$

6 $${\text{nn}} = \frac{{\left[ {{\text{NO}}_{3}^{ - } } \right]_{t2} - \left[ {{\text{NO}}_{3}^{ - } } \right]_{t1} }}{t}$$

where *m* = gross N mineralization rate [μg N (g dry soil)^−1^ day^−1^].

*c* = microbial NH_4_^+^ consumption rate [μg N (g dry soil)^−1^ day^−1^].

*i* = gross inorganic N immobilization rate [μg N (g dry soil)^−1^ day^−1^].

nm = net N mineralization rate [μg N (g dry soil)^−1^ day^−1^].

na = net ammonification rate ([μg N (g dry soil)^−1^ day^−1^].

nn = net nitrification [μg N (g dry soil)^−1^ day^−1^].

[NH_4_^+^]_*t*1_ = NH_4_^+^ concentration at *t*1 [μg N (g dry soil)^−1^].

[NH_4_^+^]_*t*2_ = NH_4_^+^ concentration at *t*2 [μg N (g dry soil)^−1^].

APE_*t*1_ = at% ^15^N excess of NH_4_^+^ pool at *t*1.

APE_*t*2_ = at% ^15^N excess of NH_4_^+^ pool at *t*2.

*t* = time difference between *t*1 and *t*2 [day].

Microbial NH_4_^+^ consumption includes microbial NH_4_^+^ immobilization and gross nitrification. Since gross nitrification was not determined in our study, which would have required labeling with ^15^NO_3_^−^, we could not calculate microbial NH_4_^+^ immobilization. Instead, we calculated gross inorganic N immobilization rates using Eq. 3. In our calculations of gross inorganic N immobilization, net mineralization and net nitrification rates we neglected possible denitrification. Moreover, we assumed that our addition of ^15^NH_4_^+^ did not change the size of the NH_4_^+^ and NO_3_^−^ pools in soil substantially.

We used a hierarchical ANOVA (type I sum of squares) to test for effects of plant species richness and functional group composition on gross N mineralization rates, microbial NH_4_^+^ consumption, gross inorganic N immobilization, net N mineralization, net ammonification and net nitrification rates. Gross N mineralization and microbial NH_4_^+^ consumption rates were square root-transformed; and net nitrification rates were box–cox power transformed ($\lambda = 1.1$) after removing the outliers to approximate normal distribution (checked with Lilliefors normality test and histograms). The residuals vs. fitted and Q–Q plots were used to check the assumption of homoscedasticity and normality of the residuals. For net N mineralization and net nitrification data, extreme outliers were removed if they deviated by more than two standard deviations from the mean (6 outliers removed from each net rates). The ANOVA was performed with block, plant species richness, and the presence/absence of each functional group as explanatory variables. All the interactions between plant species richness and presence/absence of functional groups were non-significant and thus, are not displayed in the results. The functional groups were fitted in the following sequence: legumes, grasses, tall herbs, and small herbs. The reason for fitting legumes first among the functional groups is because legumes frequently have shown the strongest effect on the N cycle. Grasses have also often shown an effect on N transformations. To avoid the collinearity between functional group richness and each functional group, a separate model was set up for functional group richness, fitted after block to test the effect of functional group richness on gross N mineralization, microbial NH_4_^+^ consumption, gross inorganic N immobilization, net N mineralization, net ammonification and net nitrification rates. Correlations between the selected variables were analyzed using Pearson’s correlations test. All the statistical analyses were carried out in R Studio (R Studio, Version 1.1.456, R Studio Inc., Boston, MA USA) with the free statistical software R 3.5.1 (R Core Team 2018).

To explain the species richness and functional groups effects that were detected in the ANOVAs, we first ran Pearson correlations between all potential explaining variables and the three considered gross N turnover rates gross N mineralization, microbial NH_4_^+^ consumption and gross inorganic N immobilization (Table S1) and then applied Structural Equation Modeling (SEM). As the goal of the SEM approach was to identify the potential mechanisms behind the significant species richness and functional group effects on gross N turnover rates according to the ANOVAs, plant species richness, legumes and small herbs were included as the exogenous variables in the SEM and the SEM was focused on gross N mineralization and microbial NH_4_^+^ consumption, because gross inorganic N immobilization was not significantly related with species or functional group richness. Including all the potential variables (total organic carbon, aboveground and belowground community biomass, soil moisture, root C:N, microbial biomass) into one SEM did not result in an adequate model fit (Fig. S1, Table S2). This was even true after removing the non-significant pathways (Fig. S2, Table S3). Therefore, according to the literature knowledge and the results of Pearson’s correlations (Table S1), the potentially mediating variables in the SEMs were chosen. We included root C:N ratio and microbial biomass C as potential mediators of the effect of plant species richness and functional groups (legumes, small herbs) on gross N mineralization and microbial NH_4_^+^ consumption rates. Root litter quality is also considered an important source for organic matter input after root turnover. We did not include microbial C:N ratio data, because microbial C:N ratio data were only available for two blocks. According to McCune and Grace (2002), the sample size for SEMs should be at least 50. Therefore, the sample size of microbial C:N data is too small for the application of SEM. Furthermore, we included a path between gross N mineralization and microbial NH_4_^+^ consumption rates to determine if microbial NH_4_^+^ processing depends on the amount of NH_4_^+^ produced. Based on the p values, the non-significant paths in the SEMs were removed from the final model. Unstandardized path coefficients for the respective SEMs are shown in Fig. S3. We used the *χ*^2^ test (> 0.05), *p* value (> 0.05), goodness of fit index (GFI > 0.9), comparative fit index (CFI > 0.9) and normed fit index (NFI > 0.9) to evaluate the model fit (Tables S2–S4). SEM was conducted using the R package “lavaan” (Rosseel 2012).

## Results

### Effects of plant diversity on gross and net N mineralization, net ammonification and net nitrification

**Fig. 1:**
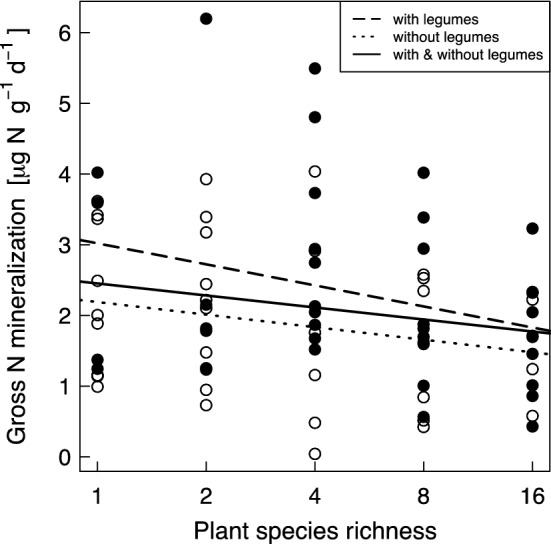
Relationship between plant species richness with/without legumes and gross nitrogen (N) mineralization. Open circles represent plots without legumes and closed circles represent plots with legumes. The regression lines are shown for illustration purpose only

Table 1 summarizes the means and ranges of all determined N turnover rates. Block had a significant effect on gross N mineralization (Table 2), net N mineralization (Table S5) and a marginally significant effect on net ammonification (Table S6). Plant species richness showed a significant negative effect on gross N mineralization rates (Table 2, Fig. 1). The mean gross N mineralization rate in the monocultures was 2.25 μg N (g dry soil) ^−1^ day^−1^ and in the sixteen plant species mixtures 1.63 μg N (g dry soil)^−1^ day^−1^, showing a decrease by 28%, which translates to a slope of a regression line of gross N mineralization rates on species number of − 0.05 μg N (g dry soil)^−1^ day^−1^ per additional species. Functional group richness had no significant effect on gross N mineralization rates (*F* = 0.13, *p* = 0.719). The presence of legumes increased gross N mineralization rates significantly (Table 2). Plant species richness was unrelated with net N mineralization, net ammonification and net nitrification (Tables S5–S7). Functional group richness was unrelated with net N mineralization (*F* = 2.64, *p* = 0.109) and net nitrification (*F* = 2.29, *p* = 0.135), but was marginally negatively related with net ammonification (*F* = 3.32, *p* = 0.073). The presence of legumes decreased net ammonification significantly (Table S6). Expectedly, net nitrification correlated significantly positively with soil 1 M KCl-extractable NO_3_^−^ concentrations from shortly before the experiment (*r* = 0.37, *p* = 0.014, NO_3_^−^ data log-transformed and 6 outliers removed).

**Table 1:** Maximum, minimum and mean values of gross and net nitrogen transformation rates

	N transformation rates [µg N (g dry soil)^−1^ day^−1^]		
	Minimum	Maximum	Mean
Gross N mineralization	0.04	6.20	2.12
Microbial ammonium consumption	− 1.81	7.24	2.43
Gross inorganic N immobilization	− 3.27	8.51	2.28
Net N mineralization	− 4.33	5.72	− 0.12
Net ammonification	− 2.57	2.13	− 0.42
Net nitrification	− 2.04	4.97	0.31

**Table 2:** Hierarchical ANOVA results showing the effects of plant species richness (SR) and presence ( +)/absence (−) of each functional group on gross nitrogen mineralization rates

Source	*df*	SS	SS (%)	*F*	*p*
**Block**	**3**	**1.45**	**10.89**	**3.15**	**0.031**
**SR**	**1**	**0.62**	**4.66**	**4.05**	**0.048↓**
**Legumes**	**1**	**0.71**	**5.33**	**4.65**	**0.035** ↑
Grasses	1	0.00	0.00	0.04	0.845
Tall herbs	1	0.26	1.95	1.68	0.199
Small herbs	1	0.31	2.33	2.05	0.157
Residuals	65	9.96			

Bold letters show significance at *p* < 0.05. Arrows indicate positive (↑) or negative (**↓**) effects

### ***Effects of plant diversity on microbial NH***_***4***_^+^***consumption and gross inorganic N immobilization***

**Fig. 2:**
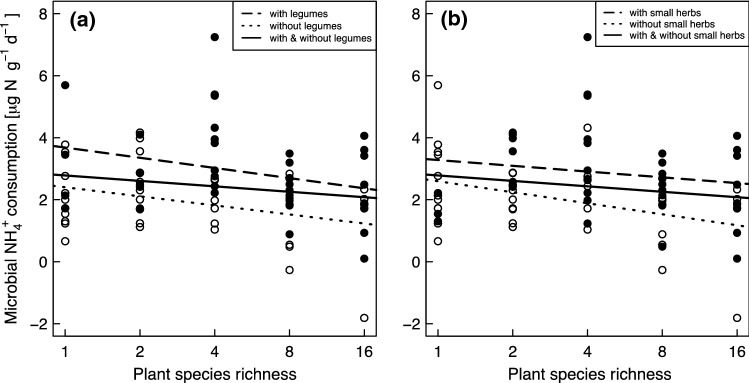
Relationship between (**a**) plant species richness with/without legumes and (**b**) plant species richness with/without small herbs and microbial ammonium (NH_4_^+^) consumption rates. Open circles represent plots without legumes/small herbs and closed circles represent plots with legumes/small herbs. The regression lines are shown for illustration purpose only

**Fig. 3:**
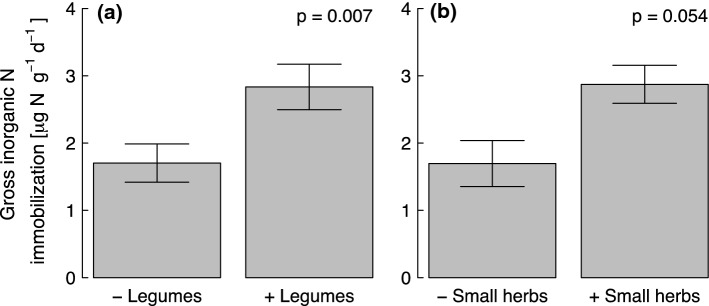
Effects of presence/absence of (**a**) legumes and (**b**) small herbs on gross inorganic N immobilization rates. *p* value is given according to the ANOVA results

Increasing plant species richness decreased the microbial NH_4_^+^ consumption rates significantly (Table 3; Fig. 2). The microbial NH_4_^+^ consumption rates were on average 2.41 and 1.87 μg N (g dry soil)^−1^ day^−1^ in the plots with one and sixteen plant species, respectively, showing a decrease by 22% that translates into a slope of a regression line of microbial NH_4_^+^ consumption rates on species number of − 0.06 µg N (g dry soil)^−1^ day^−1^ per additional species. Plant species richness was unrelated with gross inorganic N immobilization (Table 4). We did not find a significant effect of functional group richness on microbial NH_4_^+^ consumption rates (*F* = 1.84, *p* = 0.179) and gross inorganic N immobilization (*F* = 2.02, *p* = 0.160). The presence of legumes and small herbs increased microbial NH_4_^+^ consumption and gross inorganic N immobilization compared to their absence, although small herbs only had a marginally significant effect on gross inorganic N immobilization (Tables 3 and 4; Fig. 3).

**Table 3:** Hierarchical ANOVA results showing the effects of plant species richness (SR) and presence ( +)/absence (−) of each functional group on microbial ammonium consumption rates

Source	*df*	SS	SS (%)	*F*	*p*
Block	3	0.14	4.52	1.41	0.249
**SR**	**1**	**0.15**	**4.84**	**4.81**	**0.032↓**
**Legumes**	**1**	**0.50**	**16.13**	**15.64**	** < 0.001** ↑
Grasses	1	0.00	0.00	0.002	0.963
Tall herbs	1	0.04	1.29	1.17	0.283
**Small herbs**	**1**	**0.19**	**6.13**	**6.02**	**0.017** ↑
Residuals	65	2.08			

Bold letters show significance at *p* < 0.05. Arrows indicate positive (↑) or negative (**↓**) effects

**Table 4:** Hierarchical ANOVA results showing the effects of plant species richness (SR) and presence ( +)/absence (−) of each functional group on gross inorganic N immobilization rates

Source	*df*	SS	SS (%)	*F*	*p*
Block	3	14.59	5.08	1.40	0.250
SR	1	1.64	0.56	0.47	0.494
**Legumes**	**1**	**26.71**	**9.30**	**7.71**	**0.007** ↑
Grasses	1	0.13	0.05	0.04	0.845
Tall herbs	1	5.62	1.96	1.62	0.207
*Small herbs*	*1*	*13.35*	*4.65*	*3.86*	*0.054* ↑
Residuals	65	225.07			

Bold letters show significance at *p* < 0.05 and italics show significance at *p* < 0.1. Arrows indicate positive (↑) effects

### ***Effects of soil and plant community properties on gross N mineralization, microbial NH***_***4***_^+^***consumption and gross inorganic N immobilization rates***

**Fig. 4:**
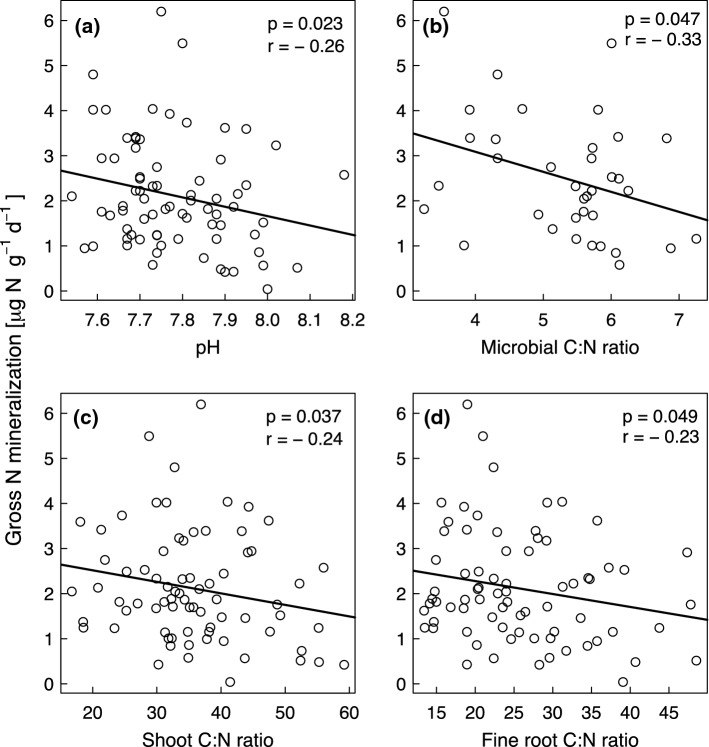
(**a**) pH, (**b**) microbial carbon to nitrogen (C:N) ratio, (**c**) shoot C:N ratio, and (**d**) fine root C:N ratio versus gross nitrogen mineralization rates. *p* and *r* values refer to results from the Pearson’s correlation test. The regression lines are shown for illustration purpose only

**Fig. 5:**
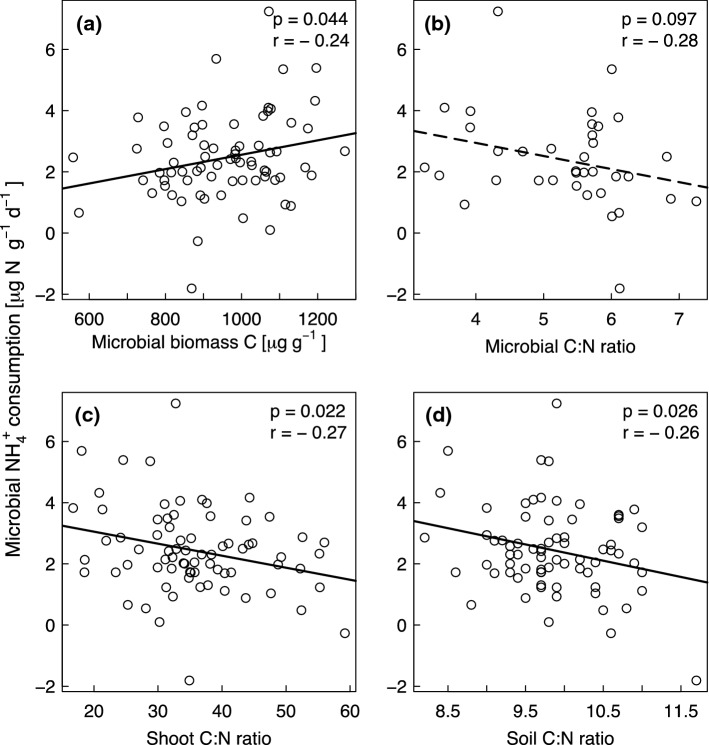
(**a**) Microbial biomass C, (**b**) microbial carbon to nitrogen (C:N) ratio, (**c**) shoot C:N ratio, and (**d**) soil C:N ratio versus microbial ammonium (NH_4_^+^) consumption rates. *p* and *r* values refer to results from the Pearson’s correlation test. The regression lines are shown for illustration purpose only. Solid lines indicate significance at *p* < 0.05 and a dashed line indicates significance at *p* < 0.1

**Fig. 6:**
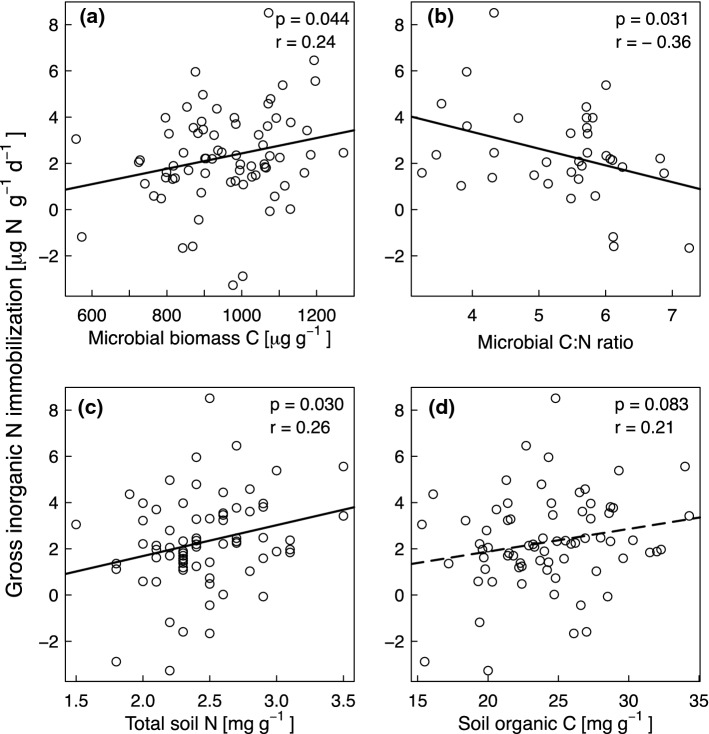
(**a**) Microbial biomass C, (**b**) microbial carbon to nitrogen (C:N) ratio, (**c**) total soil nitrogen concentrations, and (**d**) soil organic carbon concentrations versus gross inorganic N immobilization rates. *p* and *r* values refer to results from the Pearson’s correlation test. The regression lines are shown for illustration purpose only. Solid lines indicate significance at *p* < 0.05 and the dotted line indicates significance at *p* < 0.1

We tested several variables to assess the likelihood that they contributed to mechanisms by which species richness and functional group composition may have influenced gross N mineralization and microbial NH_4_^+^ consumption rates and to explore which soil and plant community properties drove gross inorganic N immobilization rates (Table S1). Soil pH showed a negative correlation with gross N mineralization rates (Fig. 4a), reflecting its influence on microbial activity. As expected, microbial biomass C had a positive relationship with microbial NH_4_^+^ consumption (Fig. 5a) and gross inorganic N immobilization rates (Fig. 6a). The microbial C:N ratios were negatively correlated with gross N mineralization rates (Fig. 4b), gross inorganic N immobilization (Fig. 6b) and microbial NH_4_^+^ consumption rates, although in the latter case only marginally significantly (Fig. 5b). We expected that lower litter quality (higher plant and soil C:N ratios) would decrease gross N mineralization and microbial NH_4_^+^ consumption rates. Supporting this hypothesis, shoot C:N (Fig. 4c) and fine root C:N ratios (Fig. 4d) had negative relationships with gross N mineralization rates and shoot C:N (Fig. 5c) and soil C:N ratios (Fig. 5d) had negative relationships with microbial NH_4_^+^ consumption rates. Furthermore, the total soil N concentrations (Fig. 6c) had significant positive and soil organic C concentrations (Fig. 6d) had marginally positive relationships with gross inorganic N immobilization rates.

**Fig. 7:**
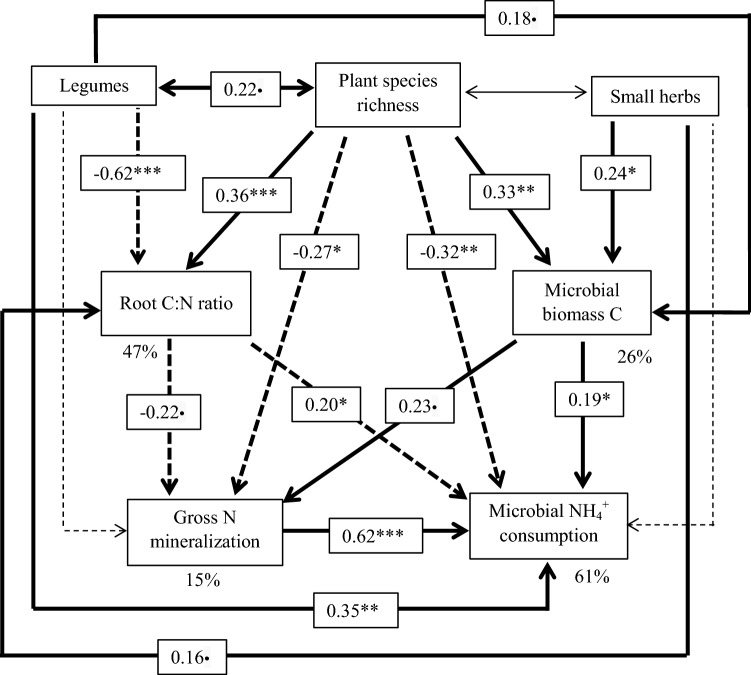
Structural equation model (SEM) to illustrate the underlying paths via which plant species richness and functional groups influenced gross N mineralization and microbial ammonium (NH_4_^+^) consumption rates. Solid and dashed thick arrows represent positive and negative significant relationships, respectively. Solid thin arrow shows a non-significant pathway. Dashed thin arrows indicate non-significant pathways that were excluded from the final model. Numbers on the arrows give standardized path coefficients with their significance indicated as ***p < 0.001, **p < 0.01, *p < 0.05, ^•^*p* < 0.01. Numbers below the variables show the percentage variation explained by corresponding variables (*R*^2^). Fit indices of the model are shown in Table S4

In the SEM set up to find possible explanations of the plant species richness and functional group effects on gross N mineralization and microbial NH_4_^+^ consumption rates (Fig. 7), the effect of plant species richness was mediated by the root C:N ratio. The root C:N ratio was the only variable out of the wealth of available data from the Jena Experiment that contributed significantly to the negative relationship of plant species richness with gross N mineralization and microbial NH_4_^+^ consumption rates. This negative effect was composed of a significantly positive effect of plant species richness on the root C:N ratio and a further significantly negative effect of the root C:N ratio on gross N mineralization and microbial NH_4_^+^ consumption rates (Fig. 7). The gross N mineralization rates had a significantly positive influence on microbial NH_4_^+^ consumption rates. The positive effect of the legumes on gross N mineralization and microbial NH_4_^+^ consumption rates was significantly related with the root C:N ratio and microbial biomass C (Fig. 7). The presence of small herbs had a positive influence on microbial NH_4_^+^ consumption rates, which was driven by increased microbial biomass C and increased root C:N ratios. There was also a direct pathway, which described a positive link between plant species richness and gross N mineralization and microbial NH_4_^+^ consumption rates via microbial biomass C. The direct path relating plant species richness with gross N mineralization and microbial NH_4_^+^ consumption rates remained significant besides the indirect effects.

## Discussion

### Plant species richness negatively affected gross N mineralization rates

The gross N mineralization rates observed in our study fall into the range of 0.32–7.09 μg N g^−1^ day^−1^ reported in the literature for comparable grasslands, i.e., natural/semi-natural grasslands with a low use intensity (Table 1; Davidson et al. 1991; Jamieson et al. 1999; Hatch et al. 2000; Wang et al. 2016). In their extensive review, Booth et al. (2005) compiled gross N mineralization rates of grasslands showing a wider range from ~ 1 to ~ 70 μg N g^−1^ day^−1^ (estimated from a figure), because their data set comprised a wider spectrum of grassland use.

We showed that increasing plant species richness reduced gross N mineralization rates (Table 2; Fig. 1), which is in contrast to our first hypothesis and the findings of West et al. (2006). Although we detected a significant negative effect of plant species richness on gross N mineralization rates, the effect was small, only explaining 5% of its variance (Table 2). Possible reasons for the contrasting results could include differences in soil type or soil pH in the study of West et al. (2006) compared to our study or to the nature of the experiment. The results of West et al. (2006) originate from a laboratory experiment, while our results were obtained from an in-situ field experiment. Cold storage of the samples before lab incubation, controlled temperature, changed nutrient supply and lack of active plant roots in lab experiments can lead to modifications of N cycling rates relative to field experiments (Arnold et al. 2008). Previous studies from the Jena Experiment have shown a significant positive effect of plant species richness on microbial activity calculated from substrate-induced respiration determined in the laboratory (Strecker et al. 2016), which also led to the expectation of enhanced gross N mineralization in species-rich plant mixtures. Our finding of a negative relationship between plant species richness and gross N mineralization is in line with the fact that plant species richness negatively affected the root decomposition in the Jena Experiment (Chen et al. 2017) and thus likely the N release rate from root turnover.

**Fig. 8:**
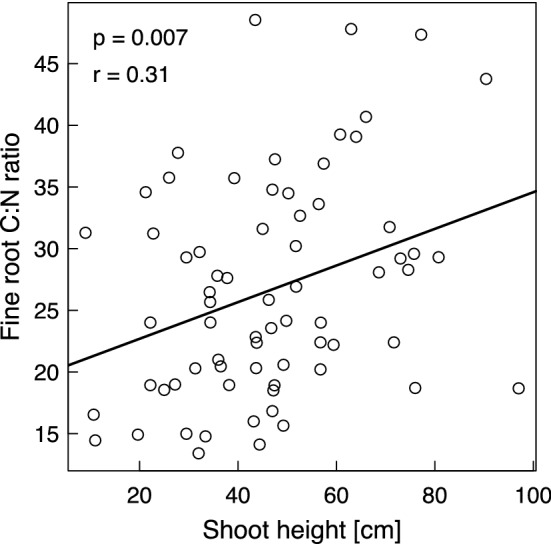
Relationship between mean regenerative shoot height (i.e., soil surface to highest flower) of the vegetation (of the year 2011) and mean fine root C:N ratios (of the year 2013)

According to the SEM, the unexpected negative relationship of species richness with gross N mineralization was related with increasing root C:N ratios with higher species richness (Fig. 7). Several reasons might explain the increasing root C:N ratios with increasing plant species richness. Guiz et al. (2015) found that N-rich legumes were increasingly replaced by small herbs that have higher root C:N ratios than legumes with increasing species richness. This is in line with reports that legumes contributed increasingly less to total biomass with increasing plant species richness (Gubsch et al. 2011; Roscher et al. 2011). Guiz et al. (2015) further speculated that increasing shoot C:N ratios with increasing plant species richness might be attributable to the dilution of plant nutrient concentrations because of the higher biomass production in species-rich mixtures, which has frequently been reported for biodiversity experiments including the Jena Experiment (Marquard et al. 2009; Fornara and Tilman 2009; Mueller et al. 2013; Ravenek et al. 2014). In the Jena Experiment, the mean plant height of a plot increased with increasing species richness (Schmidtke et al. 2010), because plants in more species-rich communities have to invest more in shoot structure in response to competition for light resulting in higher C and lower N concentrations because of the higher C:N ratios of stems than of leaves (Abbas et al. 2013; Guiz et al. 2015). Figure 8 illustrates that increasing mean shoot height translated into increasing fine root C:N ratios in the Jena Experiment. The negative impact of increasing root C:N ratios on gross N mineralization indicated by the SEM (Fig. 7) agrees well with the frequently reported finding that there is a negative relationship between the litter C:N ratio and N mineralization rates (Silver and Miya 2001; Van der Krift et al. 2001; Chen et al. 2017), because a high C:N ratio of plant tissue reflects a low litter quality (Abera et al. 2014; Zhu et al. 2014) . The fact that roots and root exudates play a vital role in regulating N mineralization (Oelmann et al. 2011) through their influence on microbial biomass and activity (Bais et al. 2006; Wang et al. 2018) further supports the important role of root properties in explaining the plant species richness effect on gross N mineralization rates. The SEM also showed another significant pathway which illustrated a positive relationship between plant species richness and gross N mineralization rates via microbial biomass C. Higher plant diversity increased microbial biomass C (Strecker et al. 2016), which further increased gross N mineralization rates (Booth et al. 2005). However, this path is marginally significant and obviously was overwhelmed by the path via the root C:N ratios.

In the Jena Experiment, the C:N ratios of aboveground biomass increased with time between 2003 and 2011. This trend was increasingly pronounced with increasing species richness (Guiz et al. 2015). Because our root C:N ratios originated from a sampling campaign 2 years after our ^15^N tracer experiment, the C:N ratios of the roots at the time of our experiment might have been lower and less differentiated between the low and the high species-richness levels. While we cannot control for this effect lacking root data from the time of our experiment, we assume that it was small. The molar C:N ratio of aboveground biomass changed from 24 to 35 (i.e., the mass-related ratio used here from 29 to 41) in 8 years, translating into a change rate of 1.45 units year^−1^. Provided that the root C:N ratios change in the same way as those of the aboveground biomass, a small change of 2.9 units (< 10% of the aboveground C:N ratio in 2011) could be expected in the 2-year lag time between our experiment and the measurement time of the root C:N ratios. A change of the root C:N ratios by 2.9 units would translate into a change of 0.09 μg N (g dry soil)^−1^ day^−1^ of the gross N mineralization rate (and of 0.1 μg N (g dry soil)^−1^ day^−1^ of the microbial NH_4_^+^ consumption rates).

We also considered the possibility that the increasing litter input with increasing species richness, which we infer from the positive plant species richness–biomass relationship, (over-)compensated the decreasing litter quality with increasing species richness. Root biomass as proxy of belowground litter input indeed showed a significant positive correlation with species richness (*r* = 0.465, *p* < 0.001) and microbial biomass (*r* = 0.34,  *p* = 0.002). However, neither aboveground nor belowground biomass correlated with gross N mineralization (Table S1), suggesting that a higher N flux with increasing litter input did not overrule the effect of the decreasing C:N ratio of both aboveground and belowground biomass. Finally, the significant negative direct path relating species richness with gross N mineralization rates suggests, that there are unknown drivers underlying this species richness effect, which we were unable to identify in spite of the wealth of available soil and plant properties.

### Plant species richness negatively affected microbial NH_4_^+^ consumption rates and had no effect on gross inorganic N immobilization rates

Microbial NH_4_^+^ consumption rates in our study fall in the range of 0.8–7.2 μg N (g dry soil)^−1^ day^−1^, earlier reported by various authors in the literature for comparable grasslands (Davidson et al. 1990; Hungate et al. 1997; Hatch et al. 2000). Again, Booth et al. (2005) reported a wider range from ~ 0.5 to ~ 80 μg N (g dry soil)^−1^ day^−1^ (estimated from a figure). We observed a negative relationship between plant species richness and microbial NH_4_^+^ consumption rates (Table 3), which is contrary to our second hypothesis. Accordingly, the expected higher N demand and tighter N cycling in species-rich than in species–poor plant mixtures did not lead to increased microbial NH_4_^+^ consumption with increasing species richness.

According to the SEM, the detected negative effect of species richness on microbial NH_4_^+^ consumption rates is partially mediated by the root C:N ratio and microbial biomass C (Fig. 7). The SEM showed that microbial NH_4_^+^ consumption rates were also affected by gross N mineralization rates. When less NH_4_^+^ was released, less NH_4_^+^ was available for microbial uptake. We assumed that the microbial C:N ratio might also play a role in mediating the effect of plant species richness on microbial NH_4_^+^ consumption because of its significant correlation with microbial NH_4_^+^ consumption (Fig. 5b). However, the microbial C:N ratio was only available for a subset of the study plots, which did not allow for including this potential mediator into the SEM. The direct path from species richness to microbial NH_4_^+^ consumption rates and the indirect one via root C:N ratios showed negative relationships. On the contrary, the indirect path between species richness and microbial NH_4_^+^ consumption rates via microbial biomass, which increased with species richness mainly because of increasing soil moisture (Lange et al. 2014) showed a positive relationship (Fig. 7). An explanation of the different signs of the three detected paths might be a positive correlation between plant species richness and microbial C:N ratio, which in turn would show a negative correlation with the microbial NH_4_^+^ consumption rates. However, we did not find any effect of plant species richness on the microbial C:N ratio in our restricted data set of two blocks (*r* = − 0.083, *p* = 0.619). Instead, we found a marginally significant negative relationship between the microbial C:N ratios and the microbial NH_4_^+^ consumption rates (Fig. 5b). Thus, we cannot support the assumption that the microbes were increasingly better supplied with N with increasing species richness and, therefore, reduced their NH_4_^+^ uptake.

Obviously, the direct and indirect (via root C:N ratios) negative effects of plant species richness on microbial NH_4_^+^ consumption again overruled its positive indirect effect (via microbial biomass). We can only speculate that the unexpected negative relationship between microbial C:N ratios and microbial NH_4_^+^ consumption rates in the Jena Experiment is attributable to the changing soil microbial community composition. In the Jena Experiment, the fungi:bacteria ratio increased with increasing species richness (Lange et al. 2014; Eisenhauer et al. 2017). The reduced microbial NH_4_^+^ consumption rates in spite of the higher microbial C:N ratios could then be attributed to the lower N demand of the fungi relative to the bacteria (Zechmeister-Boltenstern et al. 2015). This assumption is corroborated by findings that plant communities with high litter C:N ratios favor decomposition by fungi, whereas plant communities with low litter C:N ratios favor decomposition by bacteria (Wardle et al. 2004).

We tested the well-known controls of microbial NH_4_^+^ consumption rates to explain the observed negative effect of plant species richness. However, the species richness effect on microbial NH_4_^+^ consumption rates could only to a small degree be explained by our SEM (Fig. 7). We, therefore, conclude, that there must again be additional variables responsible for this negative relationship, which have not yet been studied in the Jena Experiment.

Gross inorganic N immobilization rates in our study fall in the range of 0.4–10.3 μg N (g dry soil)^−1^ day^−1^, earlier reported by various authors in the literature for comparable grasslands (Watson et al. 2000; Stockdale et al., 2002; Verchot et al. 2002; Mueller et al. 2004). The comprehensive review of Booth et al. (2005) reported a wider range from ~ 0.1 to ~ 90 μg N g^−1^ day^−1^ (estimated from a figure by combining NH_4_^+^ and NO_3_^−^ immobilization rates). Plant species richness correlated significantly positively with the 1 M KCl-extractable soil NH_4_^+^ concentrations from shortly before our pool dilution experiment (*r* = 0.30, *p* = 0.008) and significantly negatively with the 1 M KCl-extractable soil NO_3_^−^ concentrations from shortly before our pool dilution experiment (*r* = − 0.36, *p* = 0.002, NO_3_^−^ data log-transformed and 6 outliers removed). The different signs of the latter two correlations might explain that there was no relationship between plant species richness and gross inorganic N immobilization. The opposite relationships might have neutralized each other.

### Plant functional group effects on gross N mineralization, NH_4_^+^ consumption and gross inorganic N immobilization rates

The presence of legumes had a positive effect on gross N mineralization, microbial NH_4_^+^ consumption and gross inorganic N immobilization rates supporting our third hypothesis (Tables 2, 3 and 4). N_2_ fixation by legumes may increase soil N availability for other species via the mineralization of N-rich legume litter (Peoples and Craswell 1992; Spehn et al. 2002), and also via rhizodeposition and mycorrhiza (Read 1996). The presence of legumes, therefore, increased gross N mineralization, microbial NH_4_^+^ consumption and gross inorganic N immobilization rates, because legumes provide high quality litter with a low C:N ratio favoring fast decomposition rates (Abera et al. 2014). Total aboveground biomass usually increases in the presence of legumes (Tilman et al. 2001; Marquard et al. 2009), which is associated with an increased aboveground N storage in the presence of legumes (Spehn et al. 2005; Oelmann et al. 2011). Eisenhauer et al. (2010) also found increased microbial biomass C in the presence of legumes, which likely contributed to increased microbial NH_4_^+^ consumption and gross inorganic N immobilization rates. Furthermore, our result revealed a positive effect of small herbs on microbial NH_4_^+^ consumption (Table 3) and gross inorganic N immobilization rates (Table 4). Strecker et al. (2015) reported increased basal respiration and microbial biomass C in the presence of small herbs (compared to mixtures without small herbs) which increased rhizodeposition, thereby possibly leading to higher microbial NH_4_^+^ consumption or inorganic N immobilization by microorganisms.

Using plant diversity variables, we were only able to explain 10% of the variance in gross N mineralization, 27% in microbial NH_4_^+^ consumption and 14% in gross inorganic N immobilization rates (Tables 2, 3 and 4). Moreover, the well-known controls of gross N mineralization and NH_4_^+^ consumption rates (microbial C:N ratio, root C:N ratio, soil C:N ratio, shoot C:N ratio, microbial biomass C, Booth et al. 2005) individually only explained a maximum of 13% of the variance of gross N mineralization, microbial NH_4_^+^ consumption, and gross inorganic N immobilization rates (Table S1). Consequently, there must be additional unidentified controlling factors for the unexpected negative effects of plant species richness on gross N mineralization, microbial NH_4_^+^ consumption, and gross inorganic N immobilization rates. We speculate that not only the chemical quality of the roots, but also that of rhizodeposits could influence gross N mineralization, microbial NH_4_^+^ consumption and gross inorganic N immobilization. In addition to that, the influence of particular species/groups of microorganisms on the N cycle might be more than mass-proportional.

### Plant diversity effects on net N mineralization and its components net ammonification and net nitrification

Our finding of negative effects of functional group richness and presence of legumes on net ammonification (Table S6) contrasts the literature, which has up to now mainly reported positive plant diversity effects on net turnover rates (Rosenkranz et al. 2012; Mueller et al. 2013). The literature also suggested that the presence of legumes increased the net N release (Scherer-Lorenzen et al. 2003). Rosenkranz et al. (2012) stated that in the year 2006 on the same sites as in our study (The Jena Experiment) the increasing net ammonification rates with increasing species richness were related with increasing topsoil water contents. However, Fischer et al. (2019) showed that in the later course of The Jena Experiment beginning in the year 2010 and particularly 2011, the year of our experiment, the water contents decreased with increasing species richness, which they attributed to the positive effect of species richness on soil aggregation and the subsequently increased water infiltration rates. Thus, the decreasing soil water contents with increasing species richness in the year 2011 might explain the negative effect of functional group richness on net ammonification. Our finding that the presence of legumes decreased net ammonification after the effects of block and species richness had been considered is unexpected (Table S6). We attribute this to the positive effect of legumes on microbial NH_4_^+^ consumption (Table 2) and gross inorganic N immobilization (Table 3), which resulted in a smaller leftover of NH_4_^+^ in mixtures with than without legumes.

## Conclusions

Our results demonstrate that both, gross mineralization and microbial NH_4_^+^ consumption rates determined in the field unexpectedly decreased with increasing species richness, while gross inorganic N immobilization was unrelated with species richness so that we had to reject our first two hypotheses. Again unexpectedly, functional group richness had negative effects on net ammonification rates, which we attribute to the decreasing soil moisture in topsoil with increasing plant diversity in the year of our study (2011). The third hypothesis that the presence of legumes influenced gross mineralization, microbial NH_4_^+^ consumption and gross inorganic N immobilization rates positively was, however, supported by our data. This positive effect likely explained the negative effect of the presence of legumes on net ammonification.

Among the wealth of data from the Jena Experiment, only the root C:N ratio was identified to significantly reduce two of the three studied gross N turnover rates, but explained a small portion of the total variance in our structural equation model. The root C:N ratio likely increased with increasing species richness because of a species replacement effect from legumes to forbs and because of increasing competition for light which resulted in a higher mean shoot height associated with a lower C:N ratio of the above- and belowground biomass. The negative root C:N ratio effect overwhelmed a positive effect of microbial biomass on gross N mineralization and microbial N consumption. Our results illustrate that the nutrient composition of biomass mediates N turnover processes in the studied grassland ecosystem suggesting that connecting ecological stoichiometry with nutrient fluxes could be a promising avenue to better understanding the biodiversity–nutrient cycling relationship.

The significant direct effect of species richness on gross N mineralization and microbial NH_4_^+^ consumption rates, which remained in our structural equation model could not be explained based on the available data. We hypothesize that the latter is related with a changing microbial composition with increasing species richness, for which we lack data. Therefore, future experiments should be designed to elucidate the relationships between species richness, microbial community composition and N turnover rates. Generally, relating soil nutrient fluxes with microbial community composition could additionally improve our understanding of the controls of nutrient turnover in soil.

## Electronic supplementary material

Below is the link to the electronic supplementary material.

Supplementary file1 (DOCX 160 kb)

## Data Availability

The datasets generated during the current study are available from the corresponding author on reasonable request.
