# Supplementary material for: Plant diversity influenced gross nitrogen mineralization, microbial ammonium consumption and gross inorganic N immobilization in a grassland experiment
Source: Oecologia. 2020 Jul 31;193(3):731–48. doi: 10.1007/s00442-020-04717-6 (PMC7406533; doi:10.1007/s00442-020-04717-6)
Supplement: Supplementary file 1 — Supplementary file1 (DOCX 160 kb) [file 442_2020_4717_MOESM1_ESM.docx]

**Plant diversity influenced gross nitrogen mineralization, microbial ammonium consumption and gross inorganic N immobilization in a grassland experiment**

Soni Lama^1^, Andre Velescu^1^, Sophia Leimer^1*^, Alexandra Weigelt^2,3^, Hongmei Chen^2^, Nico Eisenhauer^2,3^, Stefan Scheu^4^, Yvonne Oelmann^5^, Wolfgang Wilcke^1^

^1^Institute of Geography and Geoecology, Karlsruhe Institute of Technology (KIT), Reinhard-Baumeister-Platz 1, 76131 Karlsruhe, Germany

^2^Institute of Biology, Leipzig University, Johannisallee 21, 04103 Leipzig, Germany

^3^German Center for Integrative Biodiversity Research (iDiv) Halle-Jena-Leipzig, Deutscher Platz 5e, 04103 Leipzig, Germany

^4^JF Blumenbach Institute of Zoology and Anthropology, University of Göttingen, Berliner Strasse 28, 37073 Göttingen, Germany

^5^Geoecology, University of Tübingen, Rümelinstrasse 19-23, 72070 Tübingen, Germany

*Correspondence E-mail: [sophia.leimer@kit.edu](mailto:sophia.leimer@kit.edu); Tel.: +49-721-60841618; Fax: +49-721-60843738

**Supporting information**

**Table S1** Pearson correlation coefficients for the relationships of the potential variables with gross N mineralization, microbial NH_4_^+^ consumption and gross inorganic N immobilization. Significant correlations (p<0.05) are marked in bold and marginal significant correlations (p<0.1) are marked in italics.

| **Variables** | **Gross N mineralization** | | **Microbial NH_4_^+^ consumption** | | **Gross inorganic N immobilization** | |
| --- | --- | --- | --- | --- | --- | --- |
|  | r | p | r | p | r | p |
| Microbial biomass C | 0.094 | 0.434 | **0.238** | **0.044** | **0.238** | **0.044** |
| Microbial C:N ratio | **-0.326** | **0.047** | *-0.281* | *0.097* | **-0.360** | **0.031** |
| Soil C:N ratio | -0.091 | 0.447 | **-0.262** | **0.026** | -0.019 | 0.877 |
| Shoot C:N ratio | **-0.243** | **0.037** | **-0.267** | **0.022** | -0.164 | 0.162 |
| Fine root C:N ratio | **-0.230** | **0.049** | *-0.210* | *0.072* | -0.036 | 0.758 |
| Root biomass | -0.113 | 0.336 | -0.031 | 0.796 | 0.051 | 0.665 |
| Shoot biomass | -0.137 | 0.245 | -0.078 | 0.510 | -0.094 | 0.427 |
| Total N concentration in soil | 0.066 | 0.582 | 0.126 | 0.290 | **0.256** | **0.030** |
| Total organic C concentration in soil | 0.014 | 0.907 | 0.002 | 0.985 | *0.206* | *0.083* |
| Soil moisture | -0.073 | 0.542 | 0.008 | 0.947 | -0.010 | 0.930 |
| Soil pH | **-0.264** | **0.023** | -0.059 | 0.616 | -0.098 | 0.407 |

**Table S2** Fit indices for the structural equation model in Fig. S1. P = p value, χ^2^ = chi squared, GFI = goodness of fit index, CFI = comparative fit index, NFI = normed fit index.

| Fit index | Value |
| --- | --- |
| P | 0.00 |
| χ^2^ | 73.94 |
| GFI | 0.85 |
| CFI | 0.79 |
| NFI | 0.75 |

**Table S3** Fit indices for the structural equation model in Fig. S2. P = p value, χ^2^ = chi squared, GFI = goodness of fit index, CFI = comparative fit index, NFI = normed fit index.

| Fit index | Value |
| --- | --- |
| P | 0.00 |
| χ^2^ | 47.39 |
| GFI | 0.88 |
| CFI | 0.86 |
| NFI | 0.81 |

**Table S4** Fit indices for the structural equation model in Figs. 5 and S3. P = p value, χ^2^ = chi squared, GFI = goodness of fit index, CFI = comparative fit index, NFI = normed fit index.

| Fit index | Value |
| --- | --- |
| P | 0.07 |
| χ^2^ | 10.2 |
| GFI | 0.96 |
| CFI | 0.96 |
| NFI | 0.04 |

**Table S5** Hierarchical ANOVA results showing the effects of plant species richness (SR) and presence (+) /absence (-) of each functional group on net nitrogen mineralization rates. Bold letters show significance at p < 0.05.

Source Df SS SS (%) F P

**Block 3 10.00 11.50 2.83 0.046**

SR 1 0.05 0.06 0.05 0.833

Legumes 1 3.26 3.75 2.77 0.101

Grasses 1 0.61 0.70 0.51 0.476

Tall herbs 1 0.50 0.57 0.42 0.518

Small herbs 1 0.73 0.84 0.62 0.433

Residuals 61 71.84

**Table S6** Hierarchical ANOVA results showing the effects of plant species richness (SR) and presence (+) /absence (-) of each functional group on net ammonification rates. Bold letters show significance at p < 0.05 and italics show significance at p < 0.1. Arrows indicate negative (**↓**) effects

Df SS SS (%) F P

*Block 3 5.51 8.98 2.441 0.072*

SR 1 0.08 0.13 0.112 0.739

**Legumes 1 3.65 5.95 4.852 0.031↓**

Grasses 1 0.15 0.24 0.204 0.653

Tall herbs 1 0.17 0.28 0.222 0.639

Small herbs 1 1.36 2.22 1.802 0.184

Residuals 67 50.45

**Table S7** Hierarchical ANOVA results showing the effects of plant species richness (SR) and presence (+) /absence (-) of each functional group on net nitrification rates.

Df SS SS (%) F P

Block 3 1.49 5.16 1.20 0.319

SR 1 0.37 1.28 0.89 0.350

Legumes 1 0.17 0.59 0.40 0.529

Grasses 1 0.61 2.11 1.48 0.229

Tall herbs 1 0.10 0.35 0.24 0.624

Small herbs 1 0.85 2.94 2.06 0.157

Residuals 61 25.29

Soil moisture

Legumes

Small herbs

Plant species richness

Organic carbon

Microbial biomass

Shoot biomass

Root biomass

Root C:N

Microbial NH_4_^+^ consumption

Gross N mineralization

-0.29•

0.24*

-0.29*

0.20•

0.53***

0.36**

0.43***

0.34**

0.24*

-0.60***

-0.28**

0.33**

0.19*

-0.35**

0.58***

0.36****

0.19•

0.21•

0.46**

0.15•

-0.19•

**Fig. S1** A-priori structural equation model showing the potential causal effects of plant diversity (plant species richness and presence/absence of individual functional groups) on gross N mineralization and microbial NH_4_^+^ consumption rates. Solid and dashed thick arrows represent positive and negative significant relationships, respectively. Solid thin arrows show non-significant pathways. Numbers on the arrows give unstandardized path coefficients with their significance indicated as ***p < 0.001, **p < 0.01, *p < 0.05, ^•^p < 0.01.

Soil moisture

Legumes

Small herbs

Plant species richness

Organic carbon

Microbial biomass

Shoot biomass

Root biomass

Root C:N

Microbial NH_4_^+^ consumption

Gross N mineralization

-0.27•

0.24*

-0.26*

0.16•

0.52***

0.36***

0.41***

0.34**

0.22*

-0.60***

-0.26*

0.33**

0.15•

-0.36***

0.61****

0.32***

0.20•

0.21•

-0.21*

Soil moisture

Legumes

Small herbs

Plant species richness

Organic carbon

Microbial biomass

Shoot biomass

Root biomass

Root C:N

Microbial NH_4_^+^ consumption

Gross N mineralization

-0.36*

0.21•

-0.21•

-0.26*

0.35**

0.46***

0.53***

0.36**

0.43***

0.34**

0.24*

-0.60***

-0.28**

0.33**

0.18*

-0.34**

0.15•

0.59***

Clay

0.36***

-0.21*

0.19•

0.21•

**Fig. S2** A-priori structural equation model showing the potential causal effects of plant diversity (plant species richness and presence/absence of individual functional groups) on gross N mineralization and microbial NH_4_^+^ consumption rates. Solid and dashed thick arrows represent positive and negative significant relationships, respectively. Solid thin arrows show non-significant pathway. Dashed thin arrows indicate non-significant pathways that were sequentially excluded from the final model based on the p values. Numbers on the arrows give standardized path coefficients with their significance indicated as ***p < 0.001, **p < 0.01, *p < 0.05, ^•^p < 0.01.

Gross N mineralization

0.05•

Plant species richness

Microbial NH_4_^+^ Consumption

Legumes

Small herbs

0.06•

Microbial biomass C

Root C:N ratio

0.63***

0.09**

-1.11***

-0.22*

-0.11•

0.27*

0.07*

-0.13**

0.14**

0.16•

0.70•

0.05*

0.29***

**Fig. S3** Structural equation model (SEM) to illustrate the underlying paths via which plant species richness and functional groups influenced gross N mineralization and microbial NH_4_^+^ consumption rates. Solid and dashed thick arrows represent positive and negative significant relationships, respectively. Solid thin arrow shows a non-significant pathway. Dashed thin arrows indicate non-significant pathways that were excluded from the final model. Numbers on the arrows give unstandardized path coefficients with their significance indicated as ***p < 0.001, **p < 0.01, *p < 0.05, ^•^p < 0.01.
